# Overcoming acquired resistance to HSP90 inhibition by targeting JAK-STAT signalling in triple-negative breast cancer

**DOI:** 10.1186/s12885-019-5295-z

**Published:** 2019-01-24

**Authors:** Nuramalina H. Mumin, Neele Drobnitzky, Agata Patel, Luiza Madia Lourenco, Fiona F. Cahill, Yanyan Jiang, Anthony Kong, Anderson J. Ryan

**Affiliations:** 1 Department of Oncology, University of Oxford, Oxford, UK; 2 Institute of Cancer and Genomic Sciences, University of Birmingham, Birmingham, UK

**Keywords:** Triple-negative breast cancer, TNBC, Breast cancer, Heat shock protein, HSP90, Ganetespib, Resistance, JAK, STAT

## Abstract

**Background:**

Due to the lack of effective therapies and poor prognosis in TNBC (triple-negative breast cancer) patients, there is a strong need to develop effective novel targeted therapies for this subtype of breast cancer. Inhibition of heat shock protein 90 (HSP90), a conserved molecular chaperone that is involved in the regulation of oncogenic client proteins, has shown to be a promising therapeutic approach for TNBC. However, both intrinsic and acquired resistance to HSP90 inhibitors (HSP90i) limits their effectiveness in cancer patients.

**Methods:**

We developed models of acquired resistance to HSP90i by prolonged exposure of TNBC cells to HSP90i (ganetespib) in vitro. Whole transcriptome profiling and a 328-compound bioactive small molecule screen were performed on these cells to identify the molecular basis of acquired resistance to HSP90i and potential therapeutic approaches to overcome resistance.

**Results:**

Among a panel of seven TNBC cell lines, the most sensitive cell line (Hs578T) to HSP90i was selected as an in vitro model to investigate acquired resistance to HSP90i. Two independent HSP90i-resistant clones were successfully isolated which both showed absence of client proteins degradation, apoptosis induction and G2/M cell cycle arrest after treatment with HSP90i. Gene expression profiling and pathway enrichment analysis demonstrate significant activation of the survival JAK-STAT signalling pathway in both HSP90i-resistant clones, possibly through IL6 autocrine signalling. A bioactive small molecule screen also demonstrated that the HSP90i-resistant clones showed selective sensitivity to JAK2 inhibition. Inhibition of JAK and HSP90 caused higher induction of apoptosis, despite prior acquired resistance to HSP90i.

**Conclusions:**

Acquired resistance to HSP90i in TNBC cells is associated with an upregulated JAK-STAT signalling pathway. A combined inhibition of the JAK-STAT signalling pathway and HSP90 could overcome this resistance. The benefits of the combined therapy could be explored further for the development of effective targeted therapy in TNBC patients.

**Electronic supplementary material:**

The online version of this article (10.1186/s12885-019-5295-z) contains supplementary material, which is available to authorized users.

## Background

Triple-negative breast cancer (TNBC), which accounts for about 10% of all breast cancer cases, is defined by the absence of ER (estrogen receptor) and PR (progesterone receptor) expression, together with lack of HER2 (human epidermal growth factor 2 receptor) gene amplification or protein overexpression at diagnosis. TNBC is often associated with younger age (< 50) and more common in African-American women [1–3]. The tumours are generally larger in size, more aggressive, of higher grade and have propensity of lymph node involvement [4].

Chemotherapy is the only effective systemic treatment option for TNBC patients. Although TNBC patients have a significantly higher complete response rate to neoadjuvant chemotherapy compared to other breast cancer subtypes, TNBC patients have lower rates of progression-free survival and overall survival within the first 3 years [5]. After chemotherapy, TNBC patients with residual disease have a significantly worse overall survival compared to other breast cancer subtypes displaying residual disease [5]. Due to the lack of approved targeted therapies and poor prognosis in TNBC patients, there is an urgent need to develop effective treatments for this subtype of breast cancer.

High expression of HSP90 (heat shock protein 90) is associated with poor prognosis and worse recurrence-free survival in breast cancer patients, including TNBC patients [6–8]. HSP90 is a conserved molecular chaperone that regulates the stability, activation and maturation of more than 200 client proteins including receptor tyrosine kinases (HER2, EGFR, IGF-1R, MET), transcription factors (HIF1, TP53), signalling proteins (AKT, SRC) and cell cycle regulatory proteins (CDK4, CDK6). Since HSP90 client proteins play key roles in the biological hallmarks of cancer, targeting HSP90 provides the prospect of disrupting multiple oncogenic pathways simultaneously [9–12].

HSP90 was first identified as a therapeutic target when treatment with natural product compounds, geldanamycin and radicicol, decreased the levels of oncogenic client proteins (SRC, HER2) by destabilising the HSP90-client protein complex and directing it for ubiquitin-mediated proteosomal degradation [13–15]. Several preclinical studies with HSP90 inhibitors demonstrated effective anti-cancer activities at tolerable doses, where simultaneous degradation of oncogenic client proteins and inhibition of tumour growth were observed in multiple cancer models, including ovarian, prostate and breast cancer [16–18]. HSP90 inhibitors also showed tumour selectivity as they had higher binding affinity to HSP90 from tumours compared to normal tissue, and were accumulated in tumours and cleared rapidly from blood and normal tissues in vivo [19, 20].

Ganetespib was the most advanced HSP90 inhibitor in clinical development, when it entered Phase III clinical trial in non-small cell lung cancer in combination with docetaxel (NCT01798485). Patients were given ganetespib (150 mg/m^2^) and docetaxel (75 mg/m^2^) at Day 1 and ganetespib only at Day 15 of each 3-week treatment cycle. Although the trial showed no benefit, the study was performed in unselected patients. Ganetespib is a resorcinol-based HSP90 inhibitor that has a unique triazolone moiety and lacks the benzoquinone chemical moiety, which is often associated with the dose-limiting hepatotoxicity of geldanamycin-derived HSP90 inhibitors [21–23]. Ganetespib demonstrated higher in vitro potency, greater anti-cancer activity, and more efficient distribution in tumours without causing serious toxicity (liver, cardiac and ocular) [23–25]. In TNBC, ganetespib caused the downregulation of multiple client proteins (EGFR, IGF-1R, MET and CRAF), G2/M phase cell cycle arrest, inhibition of metastasis and tumour growth [26, 27]. In clinical trials, HSP90 inhibitors have shown evidence of anti-tumour activities in patients with HER2-positive breast cancer and TNBC disease [28–31]. Despite these encouraging clinical responses, they were short-lived and not all patients responded to the treatment, suggesting that resistance to HSP90 inhibition (HSP90i) develops in breast cancer patients.

The mechanisms of resistance to HSP90i in TNBC are not yet understood. A greater understanding of these resistance mechanisms should help to identify potential predictive biomarkers for a better selection of responsive patients to HSP90i therapy and help in the development of effective therapeutics for TNBC patients. In this study, whole transcriptome profiling and a bioactive small molecule screen were performed on TNBC cells with acquired resistance to HSP90i to identify the molecular basis of their resistance and potential therapeutic approaches to overcome it.

## Methods

### Cell culture and reagents

MDA-MB-231, MDA-MB-468 and MDA-MB-453 cell lines were obtained from the American Type Culture Collection. HCC-1143, HCC-1937 and Hs578T cell lines were obtained from Dr. P McGowan (St Vincent’s University Hospital, Ireland) while BT-20 cell line was obtained from Professor Adrian Harris (University of Oxford, UK). MDA-MB-453, MDA-MB-468, MDA-MB-231, Hs578T and BT-20 cell lines were maintained in DMEM (Dulbecco’s modified Eagle medium; Life Technologies), supplemented with 10% (*v*/v) foetal bovine serum (FBS; Sigma-Aldrich), 2 mM GlutaMax (Life Technologies) and 50 U penicillin/ 50 μg/ml streptomycin (Life Technologies). HCC-1143 and HCC-1937 cell lines were maintained in RPMI-1640 medium (Life Technologies), supplemented with 10% (v/v) FBS, 2 mM GlutaMax and 50 U penicillin/ 50 μg/ml streptomycin. The cell lines were authenticated using Short Tandem Repeat (STR) analysis and tested for Mycoplasma infection using PCR, as described in [32].

Ganetespib and a 326-compound bioactive small molecule library (L1100), including 17-AAG and NVP-AUY922, were obtained from Selleckchem (USA). All stock solutions were prepared DMSO and stored at − 20 °C.

### Generation of ganetespib resistant clones

Hs578T cells were plated onto 10 cm dishes (10^6^ cells per plate) and after 3 days exposed to 30 nM ganetespib. Fresh complete culture medium with ganetespib was replaced at least once a week. After 6 weeks, colonies formed on the plates (0–10 colonies per plate). Independent colonies from separate plates were isolated using cloning cylinders (Sigma-Aldrich, #C1059). The clones were maintained in culture with the continuous presence of 30 nM ganetespib. The clones remained stably resistant to ganetespib even after culturing for 2 weeks in ganetespib-free medium.

### Cell viability assay

Cell viability was assessed using a resazurin-based assay. Cells were seeded in triplicates in 96-well plate and treated the next day with indicated compound or combination of compounds for 72 h (hr). A range of drug concentrations was used by performing serial dilutions with complete culture medium. After treatment, resazurin solution was added into each well to a final concentration of 12.5 μg/ml and incubated for 2 h. The metabolically active viable cells reduce the resazurin to highly fluorescent resorufin. The fluorescence intensity (excitation 544 nm; emission at 590 nm) was measured using a POLARstar Omega plate reader (BMG Labtech GmbH, Germany). After subtracting the average fluorescence intensity of blank (medium, no cells) from the wells, the fluorescence intensity was proportional to the number of viable cells. The percent cell viability after each treatment was normalised either to DMSO-treated control or to cells treated at the fixed concentration of compound alone for combination treatment. IC_50_ (the drug concentration that reduces cell viability to 50% of control) was calculated from three independent experiments, each with triplicates measurements, using the Origin software (OriginLab Corporation, USA).

### Western blotting

Cells were seeded in 10 cm plates and treated the next day with indicated compound or combination of compounds for 24 h. After treatment, cells including in medium were collected, washed twice with ice-cold PBS and lysed in ice-cold lysis buffer containing 50 mM Tris-HCl (Promega, USA) (pH 7.6), 137 mM NaCl (Sigma-Aldrich), 10% (*v*/v) glycerol (VWR, USA), 0.1% (v/v) Igepal (Sigma-Aldrich), 0.1% (v/v) SDS (AppliChem GmbH, Germany), 50 mM sodium fluoride (Sigma-Aldrich), 1 mM sodium orthovanadate and cOmplete protease inhibitor cocktail (Roche, Switzerland). The cell suspension was left on ice for 30 min (min) before brief sonication (twice for 10 s) with a Bioruptor® Plus (Diagenode Inc., Belgium). The cells were centrifuged at 16,100 x g for 10 min at 4 °C and supernatants containing soluble proteins were collected for subsequent experiments.

Equal concentration of protein lysates were prepared in sample buffer containing 25 mM Tris-Base (Sigma-Aldrich) (pH 6.8), 1% (*v*/v) SDS, 1 mM AccuGENE EDTA (Lonza), 2.5% (*v*/v) β-mercaptoethanol (Sigma-Aldrich) and 0.25 mg/ml Bromophenol blue (Acros organics, USA), and the prepared lysates were heated to 95 °C for 8 min. The protein lysates (30–50 μg) were resolved onto a 4–15% gradient precast TGX gel, transferred to a PVDF membrane (0.45 μM pore size, Millipore), blocked in TBS (Tris-buffered saline) with 5% (*w*/*v*) non-fat skimmed milk (Sigma-Aldrich) and probed overnight with the appropriate primary antibodies prepared in 5% (w/v) non-fat skimmed milk in TBST (TBS with 0.1% (v/v) Tween 20). The membranes were incubated with horseradish peroxidase (HRP)-conjugated secondary antibodies prepared in TBST-5% (w/v) non-fat skimmed milk, and then ECL western blotting substrate was added onto the membranes and protein signals were visualised by chemiluminescence on a ChemiDoc MP Imaging System (Bio-Rad). Antibodies used: were from Cell Signaling (total EGFR, #2232, 1:1000; total AKT #9272, 1:1000; phospho-AKT S473 #9271, 1:500; HSP90 #4877, 1:1000; HSP70 #4876, 1:500; PARP #9542, 1:2000; total STAT3; #4904, 1:1000; phospho-STAT3 Y705; #9131, 1:500), Sigma-Aldrich (β-actin, #A1978, 1:5000), Abcam (Cyclophilin B (PPIB), #ab16045, 1:5000) or Invitrogen (phospho-EGFR Y1068, #44788G, 1:500).

### Cell cycle analysis

Cells were seeded in duplicate in 10 cm plates and treated the next day with indicated compound for 24 h. 20 min before the end of treatment, cells were pulse labelled with 20 μM Bromodeoxyuridine (BrdU; Sigma-Aldrich) and protected from light. After treatment, the cells on the plate including in the medium were collected and centrifuged at 280 x g for 5 min. Cell pellets were washed with PBS and fixed by addition dropwise of ice-cold ethanol (70% *v*/v, Sigma-Aldrich) while vortexing. The cells were incubated on ice for 30 min, centrifuged at 280 x g for 5 min and resuspended in 2 M HCl (Fisher Scientific) containing 0.1 mg/ml pepsin (Sigma-Aldrich). After incubation for 20 min and centrifugation, the cell pellets were washed twice with PBS, once with PBS-2% (v/v) FBS and were later probed for 90 min with mouse anti-BrdU antibody (BD Biosciences, #347580, 1:100) prepared in PBS-2% FBS. Cells were washed once with PBS-2% FBS and incubated with goat anti-mouse Alexa Fluor® 488 secondary antibody (Life Technologies, 1:200) prepared in PBS-2% FBS for 1 h in the dark. Cells were washed once with PBS and stained with 20 μg/ml of propidium iodide (PI) (Calbiochem) prepared in PBS. Cells in S-phase (BrdU staining) and DNA content (PI staining) were assessed using FACScan Flow Cytometer (BD Biosciences) and BD CellQuest Pro software. Cell cycle distributions (G0/G1-, S- and G2/M- phases) were determined using ModFit LT (Verity Software House, USA) and FlowJo software (Tree Star Inc., USA).

### RNA sequencing

Parental Hs578T cell line and ganetespib-resistant clones were seeded in 10 cm plates and treated the next day with 30 nM ganetespib or DMSO control for 24 h. After treatment, cells were collected and RNA was isolated using the RNeasy® Plus Mini Kit (Qiagen). Duplicate samples (1 μg RNA in 30 μl) were subjected to RNA Sequencing (RNA-seq) analysis (Oxford Gene Technology) and gene expression was quantified using their analysis pipeline [33]. Briefly, mRNAs were first isolated from the RNA samples and libraries for sequencing were prepared using an RNA Sample Prep Kit (TruSeq v2, Illumina). Paired-end sequencing was performed using HiSeq2000 platform (Illumina) and the sequencing reads were mapped onto the human genome (GRCh37) using Bowtie (http://bowtie-bio.sourceforge.net/index.shtml/) and splice junctions were identified using TopHat (http://ccb.jhu.edu/software/tophat/index.shtml). The mapped reads were assembled to transcripts and aligned (Cufflinks, http://cole-trapnell-lab.github.io/cufflinks//) for differential gene expression between samples. The differential gene expressions were expressed in log2 fold change (FC) and they were considered statistical significant with q-value ≤0.05. Q-value is an adjusted *p*-value that takes into account the false discovery rate within the positive test. Pathway enrichment analysis was performed on differentially expressed genes in the cells using MetaCore (https://portal.genego.com, accessed August 2016) (Thomson Reuters, USA).

### Enzyme-linked immunosorbent assay (ELISA)

Cells were seeded in 96-well plate and treated the next day with 30 nM ganetespib or DMSO control for 24 h. After treatment, the media from the wells were collected and enzyme-linked immunosorbent assay (ELISA) was performed to measure the amount of IL6 in the media using the Quantikine ELISA Human IL6 kit (R&D Systems).

### Bioactive small molecule screen

A library (*n* = 328) containing 326 bioactive small molecule compounds (Selleck, L1100), was used in the screen. The library was distributed into four 96-well plates and each plate also contained wells with DMSO. Ganetespib-resistant clone CR3 cells were seeded into 96-well plates and treated the next day with the indicated compounds (final concentration of 1 μM), in the absence or presence of 10 nM ganetespib for 72 h. Each plate was set up in duplicate and the entire screen was performed twice. Cell viability (resazurin-based assay) was the screening end point.

The percentage cell viability after each treatment of compound was first calculated relative to the average DMSO-treated control in each plate. The average percentage cell viability from the two independent runs performed in duplicates was then normalised to the overall mean percentage of cell viability from the entire screen for the respective cell line condition (absence or presence of ganetespib). In order to compare the sensitivity (Z-score) of each compound between the two conditions, the log value of the cell viability to each compound between the cell lines was calculated and normalised to the standard deviation of the entire screen. Z-score ≥ 2 showed that the compound was selectively cytotoxic in CR3 with the presence of ganetespib while Z-score ≤ − 2 showed that the compound was selectively cytotoxic in CR3 with the absence of ganetespib.

### Statistical analysis

GraphPad Prism® 7 (GraphPad Software, USA) was used for all statistical analyses and to generate graphs including heatmaps. For data comparison between cell lines and treatment conditions, two-way analysis of variance (ANOVA) was used. For comparison between two groups, Student’s t-test was used. Statistical significance of *p*-values ≤0.05, ≤ 0.01, ≤ 0.001, ≤0.0001 were represented as *, **, ***, **** respectively.

## Results

### In vitro model of acquired HSP90i-resistant TNBC cell line

**Fig. 1:**
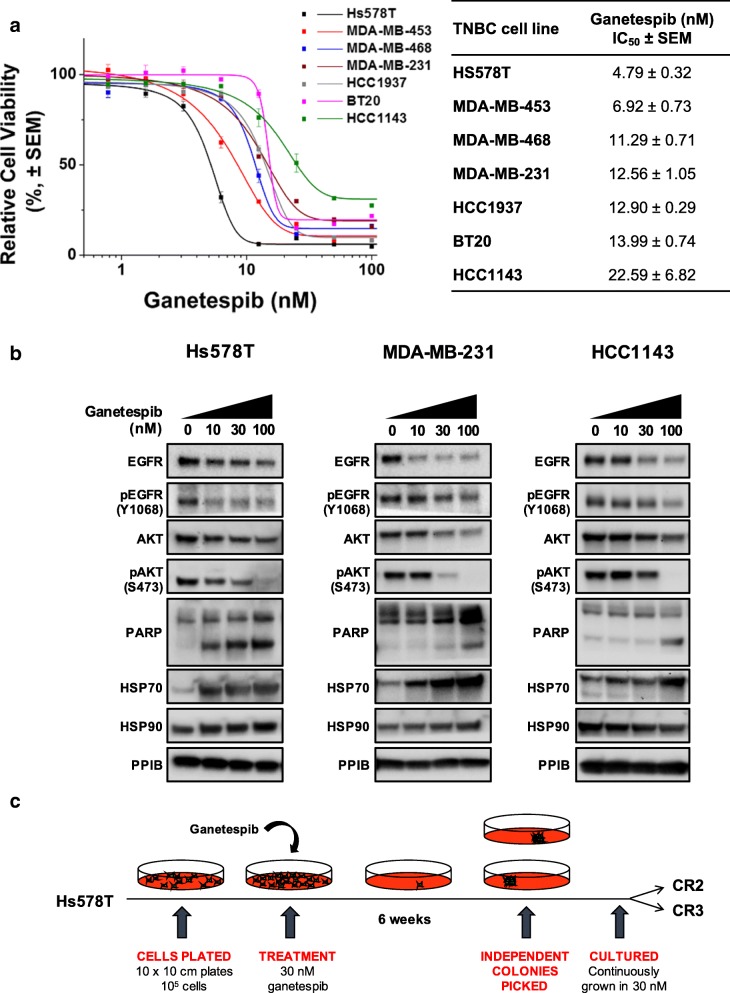
Anti-cancer activities of HSP90 inhibition in TNBC cells. **a** Differential sensitivity of ganetespib in TNBC cell lines. A panel of seven TNBC cell lines (HS578T, MDA-MB-453, MDA-MB-468, MDA-MB-231, HCC 1937, BT20 and HCC1143) were treated with increasing concentrations of ganetespib for 72 h and subjected to resazurin-based cell viability assay. Cell viability (%) for each treatment was expressed relative to the DMSO-treated control. Representative graph from at least three independent experiments each performed in triplicate and error bars indicate SEM. IC_50_ (the drug concentration that reduces cell viability to 50% of control) was calculated from the three independent experiments for each cell line. **b** Downregulation of HSP90 client proteins and induction of apoptosis by ganetespib. HS578T, MDA-MB-231 and HCC1143 cell lines were treated with increasing concentrations of ganetespib (10, 30 and 100 nM) for 24 h. Protein lysates of the treated cells were subjected to western blotting analysis and blotted with the indicated antibodies. Cleaved PARP was used to assess apoptosis and Hsp70 was used as a pharmacological marker for inhibition of Hsp90 activity. PPIB was used as loading control. **c** Generation of HSP90i-resistant clones. Hs578T cells (10^5^) were plated and treated with 30 nM of ganetespib. After 6 weeks, colonies started to form and two independent clones (CR2 and CR3) were picked from different plates

Ganetespib is highly potent in a panel of seven TNBC cell lines, with IC_50_ values range of 4 nM to 30 nM (Fig. 1a). Exposure to increasing concentrations of ganetespib resulted in a dose-dependent downregulation of HSP90 client proteins (EGFR and AKT) and their phosphorylation state in the TNBC cell lines (Fig. 1b). As expected, a pharmacodynamic marker of HSP90i, HSP70 expression was upregulated in the cell lines after the treatment. An increased level of cleaved PARP expression was also observed in these cells indicating an induction of apoptosis after HSP90i. Apoptosis induction was associated with ganetespib sensitivity in these cells, where the most sensitive TNBC cell line, Hs578T cells, showed higher induction of apoptosis after ganetespib treatment compared to MDA-MB-231 and HCC1143 cells. These data demonstrate that HSP90i has anti-cancer activity in TNBC in vitro.

**Fig. 2:**
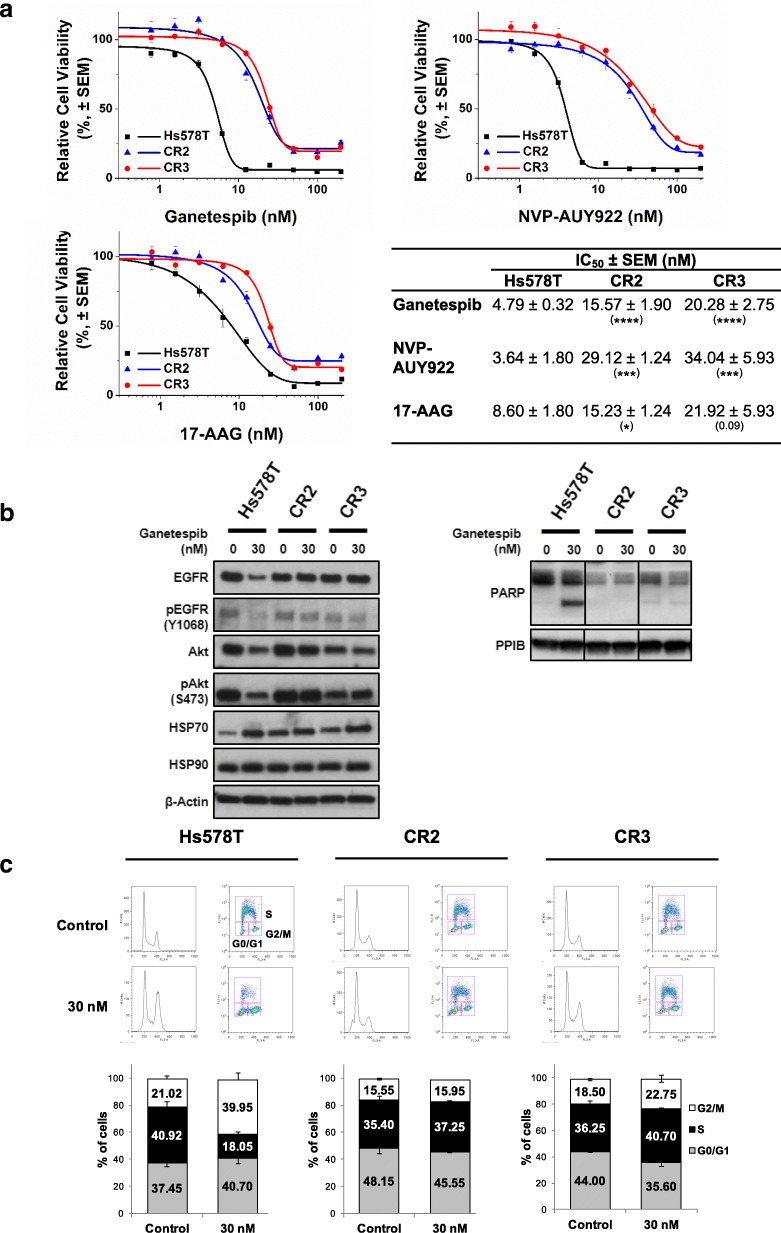
Resistance to HSP90i in CR2 and CR3. **a** Cross-resistance to HSP90i. Hs578T, CR2 and CR3 cells were treated with increasing concentrations of either ganetespib, NVP-AUY922 or 17-AAG for 72 h and subjected to resazurin-based cell viability assay. Cell viability (%) for each treatment was expressed relative to the DMSO-treated control. Representative graph from at least three independent experiments each performed in triplicate and error bars indicate SEM. The table represents the IC_50_ values of compounds in the cell lines. The IC_50_ values in respective clones were compared with the values in the parental Hs578T cells. *, *** and **** indicate *p*-value ≤0.05, ≤0.001 and ≤ 0.0001 respectively; by Student’s t-test. **b** Absence of downregulated expression of HSP90 client protein and induction of apoptosis. Hs578T, CR2 and CR3 cells were treated with 30 nM ganetespib for 24 h. Lysates were subjected to western blotting analysis and blotted with indicated antibodies. Level of cleaved PARP was also determined to assess apoptosis. PPIB and β-actin were used as loading controls. Representative images from two experiments are shown. **c** G2/M cell cycle arrest observed in parental Hs578T cells only. Hs578T, CR2 and CR3 cells were treated with 30 nM ganetespib for 24 h and pulse labelled with BrdU for 20 min. Cells were stained with propidium iodide and anti-BrdU antibody before analysed by flow cytometry. The top images represent the cell population with BrdU staining and PI staining, which consist of G0/G1- (bottom left), G2/M- (bottom right) and S-phase. The graph represents the mean percentage of cells in each phase of cell cycle from two independent experiments. Error bars indicate SEM

To investigate the mechanisms of resistance to ganetespib, two independent clones were isolated by exposing the most sensitive cell line Hs578T, with IC_50_ value of 4.79 ± 0.32 nM, to a high concentration of ganetespib (30 nM) for 6 weeks (Fig. 1c). Both clones CR2 and CR3 were resistant to ganetespib with IC_50_ values 4- to 5- fold higher than parental Hs578T cells (15.57 ± 1.90 nM and 20.28 ± 2.75 nM respectively) (Fig. 2a). Interestingly, they also showed cross-resistance to other HSP90i; NVP-AUY922 and 17-AAG with similar or higher IC_50_ (Fig. 2a), further suggesting that both clones have acquired resistance to HSP90i.

Compared to the parental Hs578T cells, the levels of total and phosphorylated EGFR and AKT proteins in the HSP90i-resistant clones were maintained and there was absence of apoptosis induction after exposure to ganetespib (Fig. 2b). However, upregulation of HSP70 expression levels were observed in the HSP90i-resistant clones after treatment, which further suggested that the compound was pharmacologically active but the clones were able to escape the inhibitory effects of ganetespib. Flow cytometry analysis also demonstrated that the HSP90i-resistant clones were able to escape the G2/M cell cycle arrest caused by HSP90i. Only the parental Hs578T cells but not the HSP90i-resistant CR2 and CR3 clones that showed a reduction in the BrdU-labelled S-phase cell population and an increase in the BrdU-labelled G2/M-phase cell population after ganetespib treatment (Fig. 2c).

### Upregulation of JAK-STAT signalling pathway after acquiring resistance to HSP90i

**Fig. 3:**
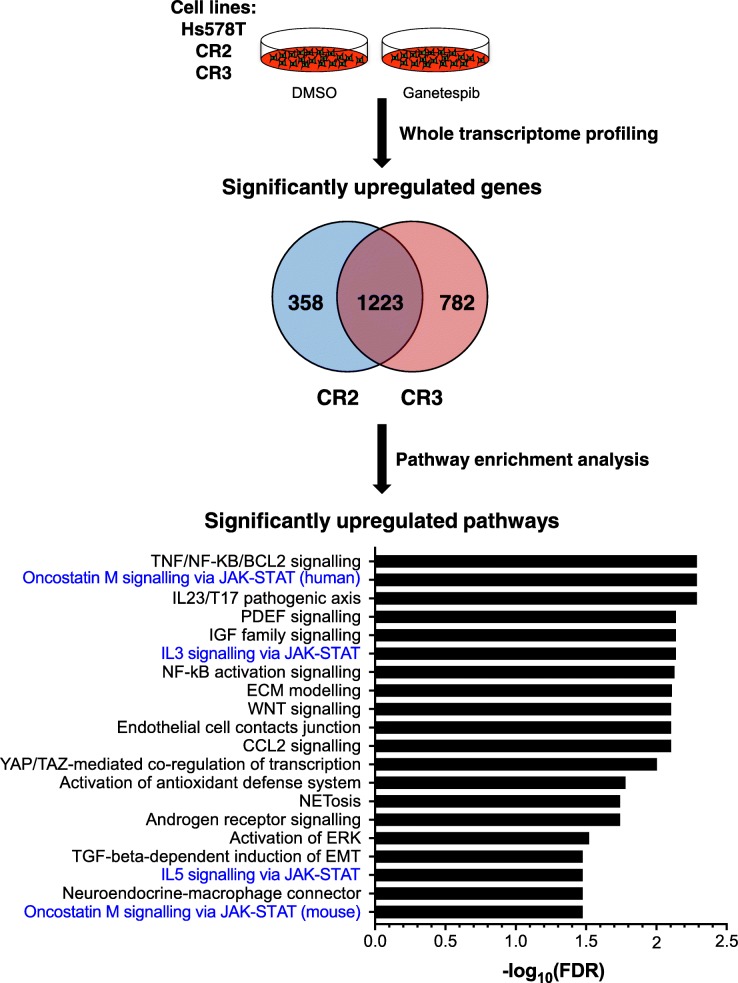
Differential RNA expression in HSP90i-resistant clones compared to parental Hs578T cells. RNA samples from DMSO-treated and ganetespib-treated Hs578T, CR2 and CR3 cells were analysed for whole transcriptome profiling with RNA-sequencing. Differential gene expression analyses were performed between DMSO-treated parental Hs578T cells with either DMSO-treated CR2 or CR3 and the significantly upregulated genes observed in these clones were mostly overlapping. Pathway enrichment analysis using Metacore™ was performed on the significantly upregulated overlapping genes. The graph represents the top 20 most significantly upregulated pathways in the HSP90i-resistant clones, with FDR (false discovery rate) value < 0.05. Pathways highlighted in blue are linked to JAK-STAT signalling

**Fig. 4:**
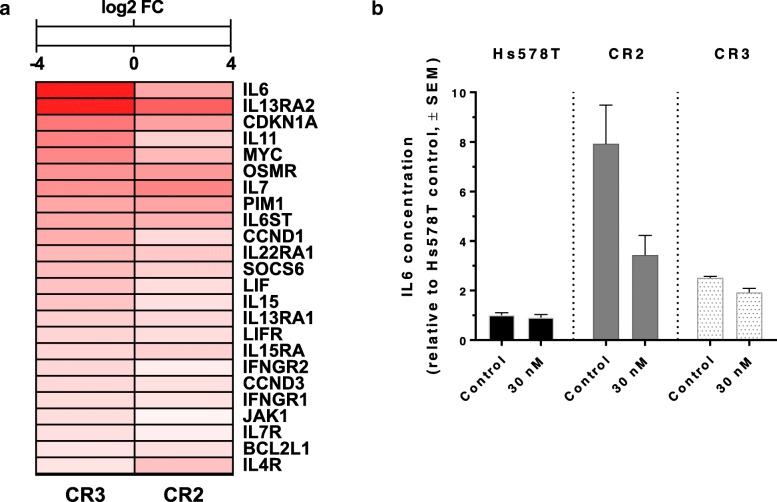
Upregulation of JAK-STAT signalling pathway in HSP90i-resistant clones. **a** Upregulation of genes involved in JAK-STAT signalling pathway. Heatmap represents the significantly upregulated genes involved in the JAK-STAT signalling pathway in HSP90i-resistant clones CR2 and CR3 compared to parental Hs578T cells. The colour scale indicates the significant log2 FC (q-value ≤0.05) in gene expression for individual clones compared to Hs578T cells from blue (− 4, downregulation) to red (+ 4, upregulation). **b** Increased IL6 expression in HSP90i-resistant clones CR2 and CR3. Media from Hs578T, CR2 and CR3 cells after treatment with ganetespib (30 nM) for 24 h were subjected to the ELISA assay in order to measure the IL6 present in the media. The graph represents the mean IL6 concentration relative to DMSO-treated Hs578T cells from two independent experiments each performed in duplicate. Error bars indicate SEM. IL6 levels were significantly higher in CR2 and CR3 than Hs578T cells, where *p* values < 0.01 by two-way ANOVA with cell line and ganetespib treatment as factors. Ganetespib treatment did not significantly affect IL6 levels in Hs578T, CR2 or CR3 cells

In order to obtain a global signature of differentially expressed genes in TNBC cells after acquiring resistance to ganetespib, whole transcriptome profiling with RNA-seq was performed in the parental Hs578T cells and both HSP90i-resistant clones CR2 and CR3 (Fig. 3). Differential gene expression analysis showed that more than 1000 transcribed genes were significantly upregulated in the HSP90i-resistant clones when compared to the parental Hs578T cells. Most of the upregulated genes were overlapping between the two clones, suggesting similar mechanisms of resistance to HSP90i. Pathway enrichment analysis on the significantly upregulated genes revealed a diverse activation of signalling pathways that could potentially mediate acquired resistance to ganetespib in the HSP90i-resistant clones, notably 4 out of the top 20 enriched pathways were linked to Janus kinase-signal transducer and activator of transcription (JAK-STAT) signalling pathway (Fig. 3 and Additional file 1: Table S1). Several genes of the JAK-STAT signalling pathway were upregulated in the HSP90i-resistant clones, including cytokine IL6 and oncogenic transcription factor MYC (Fig. 4a). ELISA assays showed that the medium of both HSP90i-resistant clones expressed significantly higher levels of IL6 compared to the parental Hs578T cells, either in the absence or presence of ganetespib (Fig. 4b). These data demonstrate that activation of the JAK-STAT pathway, possibly mediated by autocrine IL6 signalling, is associated with acquired resistance to HSP90i in TNBC cells. Of interest, pSTAT3 levels correlated with intrinsic resistance to HSP90i in TNBC cells used in this study (Additional file 2: Figure S1).

### Increased cytotoxicity of HSP90i with combined inhibition of JAK-STAT signalling pathway

**Fig. 5:**
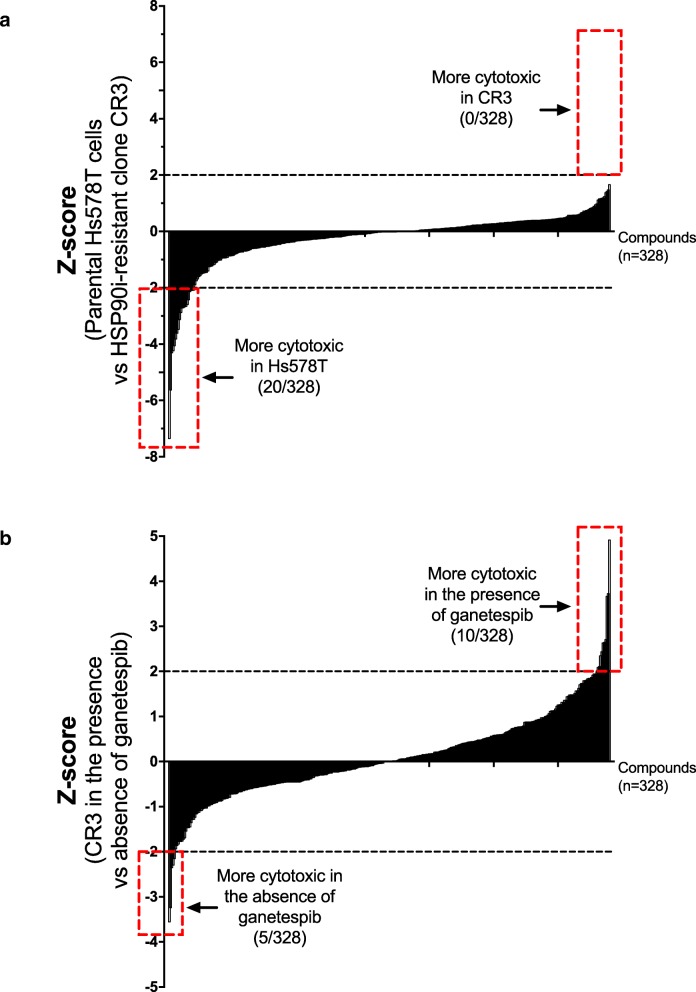
Differential sensitivity to compounds in parental Hs578T cells and HSP90i-resistant clone CR3. **a** Comparison between parental Hs578T cells and CR3. A screen with a 328-compound bioactive small molecule library was performed on the parental Hs578T cells and HSP90i-resistant clone CR3. The scatter plot represents the Z-scores comparing the sensitivity for individual compounds in Hs578T and CR3 cells. The Z-scores were ranked from compound that was least sensitive to most sensitive in CR3 when compared with Hs578T cells. Z-score ≥ 2 represents compounds that were selectively cytotoxic to CR3 compared with Hs578T cells while Z-score ≤ − 2 represents compounds that were selectively cytotoxic to Hs578T cells compared with CR3. **b** Comparison between the presence and absence of ganetespib in CR3. The screen on CR3 was also performed in the presence of ganetespib. The scatter plot represents the Z-scores comparing the sensitivity for individual compounds in CR3, in the presence and absence of ganetespib. The Z-scores were ranked from compound that was least sensitive to most sensitive in CR3, in the presence of ganetespib. Z-score ≥ 2 represents compounds that were selectively cytotoxic to CR3 in the presence of ganetespib while Z-score ≤ − 2 represents compounds that were selectively cytotoxic to CR3 in the absence of ganetespib

In order to identify potential novel targets for overcoming acquired resistance to ganetespib in TNBC, a screen with a 328-compound bioactive small molecule library was performed on the parental Hs578T cell line and HSP90i-resistant clone CR3. The library (*n* = 328) covered a broad range of established targets in cancer therapy, which were mostly approved for clinical use, or in late stage clinical trial or preclinical development. CR3 cells did not show selective sensitivity (Z-score ≥ 2) to any compounds when compared with the parental Hs578T cells, but showed selective resistance (Z-score ≤ − 2) to 20/328 compounds, which were mainly targeting proteins involved in proliferation and cell cycle checkpoints (Fig. 5a and Additional file 3: Table S2).

**Fig. 6:**
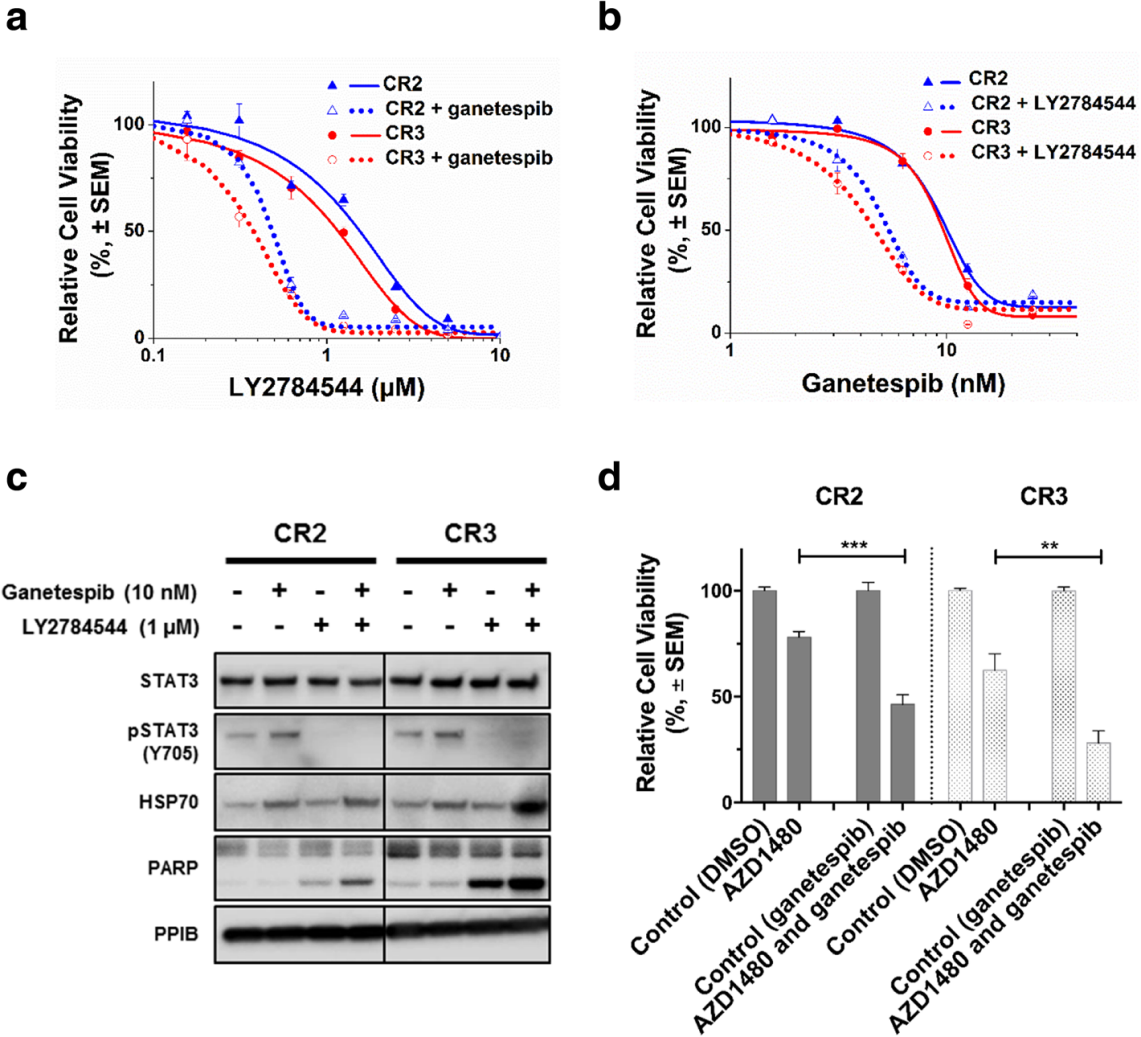
Synergistic effects after combined inhibition of HSP90 and JAK2. **a** Increased sensitivity to JAK2 inhibition. HSP90i-resistant clones CR2 and CR3 were treated with increasing concentrations of a JAK2 inhibitor, LY2784544 alone or in combination with 10 nM ganetespib for 72 h and subjected to resazurin-based cell viability assay. Cell viability (%) for each treatment was expressed relative to either DMSO-treated or ganetespib only-treated control for combination treatment. Representative graph from three independent experiments each performed in triplicates and error bars indicate SEM. **b** Increased sensitivity to HSP90i. HSP90i-resistant clones CR2 and CR3 were treated with increasing concentrations of ganetespib alone or in combination with 1 μM LY2784544 for 72 h and subjected to resazurin-based cell viability assay. Cell viability (%) for each treatment was expressed relative to either DMSO-treated or LY2784544 only-treated control for combination treatment. Representative graph from three independent experiments each performed in triplicates and error bars indicate SEM. **c** Inhibition of JAK-STAT signalling pathway and induction of apoptosis after combined treatment. HSP90i-resistant clones CR2 and CR3 were treated with ganetespib (10 nM), LY2784544 (1 μM) or in combination for 24 h. Protein lysates were analysed by western blotting and blotted with pSTAT3 (Y705), which is a downstream protein of the JAK signalling pathway. Levels of cleaved PARP and HSP70 were also determined to assess apoptosis and pharmacodynamic marker for HSP90i respectively. PPIB was used as loading control. **d** Increased cytotoxicity after combined treatment. HSP90i-resistant clones CR2 and CR3 were also treated with another JAK2 inhibitor, AZD1480 (2 μM) alone or in combination with ganetespib (10 nM) for 72 h and subjected to resazurin-based cell viability assay. The graph represents the overall mean cell viability (%) relative to DMSO-treated or ganetesib-treated control from two independent experiments each performed in triplicates. Error bars indicate SEM. The sensitivity to AZD1480; in the absence or presence of ganetespib were compared in the HSP90i-resistant clones. ** and *** indicate *p* values ≤0.01 and ≤ 0.001 respectively; by Student’s t-test

The screen with HSP90i-resistant clone CR3 was also carried out, in the absence or presence of ganetespib (10 nM) to identify compounds with selective combination effects. CR3 cells showed selective sensitivity (Z-score ≥ 2) to 10/328 compounds, in the presence of ganetespib. In contrast 5/328 compounds were selectively cytotoxic to CR3 in the absence of ganetespib (Z-score ≤ − 2) (Fig. 5b and Additional file 4: Table S3). LY2784544, which is an inhibitor of the Janus Kinase 2 (JAK2), was among the most significant hits from the screen upon retesting in CR3 as well as in the other HSP90i-resistant clone CR2. The IC_50_ values of LY2784544 in CR2 and CR3 cells were significantly decreased by 3.5- and 2.9- fold respectively, in the presence of ganetespib (Fig. 6a). There was also a significant decrease in IC_50_ values of ganetespib in CR2 and CR3 cells by 2.3- and 2.7- fold respectively, with the presence of LY2784544 (Fig. 6b).

In both HSP90i-resistant clones, western blotting analysis showed that LY2784544 treatment alone or in combination caused a marked reduction in the expression levels of pSTAT3 (Y705), which is downstream of JAK (Fig. 6c) confirming inhibition of JAK-STAT signalling pathway by LY2784544. Combined treatment of ganetespib and LY2784544 induced increased apoptosis and further upregulation of HSP70 expression in the HSP90i-resistant clones, suggesting an increase in cytotoxic activity of HSP90i with JAK2 inhibition despite prior acquired resistance to HSP90i (Fig. 6c). Combined treatment with another JAK2 inhibitor, (AZD1480) also showed significantly increased sensitivity in both HSP90i-resistant clones (Fig. 6d). These data further suggest that the combined inhibition had a synergistic effect on the HSP90i-resistant clones, despite prior acquired resistance to HSP90i.

## Discussion

Targeting HSP90 is a promising approach for the development of novel therapeutics for TNBC patients, a subtype of breast cancer with poor prognosis and lack of approved targeted therapies. In accordance with previous reports in TNBC [26, 27], we demonstrate that HSP90i using ganetespib caused inhibition of cell viability, downregulation of client proteins, induction of apoptosis and G2/M cell cycle arrest in TNBC.

Resistance to targeted therapies remains a major challenge in the treatment of cancer patients [34]. Increased expression of HSP70 is associated with reduced sensitivity to HSP90i in prostate and colon cancer cells [35, 36]. Reduced expression of NQO1 is associated with resistance to geldanamycin-based HSP90i such as 17-AAG and 17-DMAG, but not to structurally unrelated HSP90i in glioblastoma, oesophageal and breast cancer cells [37–39]. Glucuronidation via increased UGT1A expression levels is associated with resistance to resorcinol-based HSP90i such as ganetespib in colorectal cancer cells [40, 41]. Loss in MCL dependency is associated with resistance to HSP90i in cancer cells and they require a combined treatment with inhibition of BCL family proteins for induction of apoptosis [42].

The mechanisms of resistance to HSP90i in TNBC is not yet fully known. In this study, two independent HSP90i-resistant clones were established as an in vitro model to investigate the underlying mechanisms to HSP90i resistance in TNBC cells. The clones showed significant resistance to ganetespib and cross-resistance to other HSP90i compared to parental cells. Gene expression profiling and pathway analysis on the HSP90i-resistant clones suggested that activation of the survival JAK-STAT signalling pathway, possibly through autocrine IL6 signalling, might be involved in the acquired resistance to HSP90i in TNBC cells. The drug screen also suggested that combined inhibition of HSP90 and the JAK-STAT signalling pathway was able to increase the cytotoxic activities of HSP90i and overcome the acquired resistance to HSP90i in the cells. A previous study in myeloproliferative cells also showed that combination of HSP90i (NVP-AUY922) and JAK2 inhibitor (TG101209) induced the downregulation of JAK2 signalling proteins and apoptosis [43].

The JAK-STAT signalling pathway is involved in promoting oncogenesis, including cell proliferation, invasion, inflammation and immune response [44, 45]. Feedback activation of STAT3 causes resistance to various targeted therapies (HER2, EGFR, MEK) and chemotherapies by restoring cell survival and proliferation [46, 47]. Increased levels of IL6 induce the expression of anti-apoptotic proteins that inhibit apoptosis and promote proliferation in cells, which in turn are associated to mediate resistance to chemotherapy in prostate, breast and lung cancer cells [48–51]. Interestingly, the HSP90i-resistant clones in our study were also resistant to certain chemotherapies (adriamycin and mitoxantrone), suggesting that the upregulated JAK-STAT signalling pathway might also be associated with chemo-resistant in the TNBC cells.

## Conclusions

This study demonstrates that the upregulated JAK-STAT survival signalling pathway is associated with acquired resistance to HSP90i in TNBC cells and that combined therapy with JAK inhibition could overcome this resistance. Our data suggest the possibility that identifying tumours with low activity of JAK-STAT signalling pathway could potentially be used for the selection of cancer patients that would most likely benefit to ganetespib.

## Additional files

Additional file 1: **Table S1.** The top 20 most significantly enriched pathways seen in HSP90i-resistant clones. Pathway enrichment analysis performed on differentially expressed genes in parental and HSP90i-resistant Hs578T cells using MetaCore (https://portal.genego.com). (DOCX 13 kb)

Additional file 2: **Figure S1.** Positive correlation of pSTAT3 expression with HSP90i sensitivity. A scatter plot displaying the relationship between ganetespib sensitivity (IC_50_) and pSTAT3 expression in the panel of TNBC cell lines. (DOCX 25 kb)

Additional file 3: **Table S2.** Compounds that were selectively cytotoxic to parental Hs578T cells compared with HSP90i-resistant clone CR3. Cell viability was assessed after 72 h exposure to a 326-compound small molecule library (1 μM each compound. SELLECK). Z-scores ≤ − 2 identified compounds that were selectively cytotoxic to parental Hs578T cells. (DOCX 13 kb)

Additional file 4: **Table S3.** Compounds that were differentially cytotoxic to HSP90i-resistant clone CR3 in the presence of ganetespib. Cell viability was assessed after 72 h exposure to a 326-compound small molecule library (1 μM each compound. SELLECK) in the presences or absence of ganetespib (10 nM). Z-scores ≤ − 2 identified compounds that were selectively cytotoxic CR3 cells in the absence of ganetespib, Z-scores ≥2 identified compounds that were selectively cytotoxic CR3 cells in the presence of ganetespib. (DOCX 23 kb)

## Abbreviations

ANOVA: Analysis of variance
BrdU: Bromodeoxyuridine
DMSO: Dimethyl sulfoxide
EGFR: Epidermal growth factor
ELISA: Enzyme-linked immunosorbent assay
ER: Estrogen receptor
FBS: Foetal bovine serum
FC: Fold change
FDR: False discovery rate
HER2: Human epidermal growth factor 2 receptor
HSP90: Heat shock protein 90
HSP90i: HSP90 inhibition
JAK: Janus Kinase
PCR: Polymerase chain reaction
PI: Propidium iodide
PR: Progesterone receptor
RNA-seq: RNA sequencing
STAT: Signal transducer and activator of transcription
TBS: Tris buffered saline
TNBC: Triple-negative breast cancer
