# Supplementary material for: Overcoming acquired resistance to HSP90 inhibition by targeting JAK-STAT signalling in triple-negative breast cancer
Source: BMC Cancer. 2019 Jan 24;19:102. doi: 10.1186/s12885-019-5295-z (PMC6345040; doi:10.1186/s12885-019-5295-z)
Supplement: Supplementary file 1 — Table S1. The top 20 most significantly enriched pathways seen in HSP90i-resistant clones. Pathway enrichment analysis performed on differentially expressed genes in parental and HSP90i-resistant Hs578T cells using MetaCore (https://portal.genego.com). (DOCX 13 kb) [file 12885_2019_5295_MOESM1_ESM.docx]

**Table S1: The top 20 most significantly enriched pathways seen in HSP90i-resistant clones**

| **Pathway maps** | **Fraction of genes** | **p-value** | **FDR** |
| --- | --- | --- | --- |
| Apoptosis and survival: Anti-apoptotic TNFs/NF-kB/Bcl-2 pathway | 10/42 | 1.09E-05 | 5.15E-03 |
| Immune response: Oncostatin M signalling via JAK-STAT in human cells | 7/20 | 1.52E-05 | 5.15E-03 |
| Role of IL-23/ T17 pathogenic axis in psoriasis | 11/54 | 1.97E-05 | 5.15E-03 |
| Development: PEDF signalling | 10/49 | 4.63E-05 | 7.27E-03 |
| IGF family signalling in colorectal cancer | 11/60 | 5.54E-05 | 7.27E-03 |
| Immune response: IL-3 signalling via JAK-STAT, p38, JNK and NF-kB | 14/93 | 5.55E-05 | 7.27E-03 |
| Signal transduction: NF-kB activation pathways | 51/10 | 6.64E-05 | 7.45E-03 |
| Cell adhesion: ECM remodelling | 10/52 | 7.90E-05 | 7.76E-03 |
| Development: WNT signalling pathway | 10/53 | 9.36E-05 | 7.88E-03 |
| Cell adhesion: Endothelial cell contacts by junctional mechanisms | 7/26 | 1.02E-04 | 7.88E-03 |
| Immune response: CCL2 signalling | 10/54 | 1.10E-04 | 7.88E-03 |
| Development: YAP/TAZ-mediated co-regulation of transcription | 10/56 | 1.52E-04 | 9.93E-03 |
| Oxidative stress: Role of Sirtuin1 and PGC1-alpha in activation of antioxidant defence system | 10/60 | 2.74E-04 | 1.66E-02 |
| NETosis in SLE | 7/31 | 3.36E-04 | 1.81E-02 |
| Androgen receptor activation and downstream signalling in Prostate cancer | 14/110 | 3.46E-04 | 1.81E-02 |
| Development: Activation of ERK by ACM1, ACM3 and ACM5 | 8/44 | 6.12E-04 | 3.00E-02 |
| Development: TGF-beta-dependent induction of EMT via SMADs | 7/35 | 7.38E-04 | 3.34E-02 |
| Immune response: IL-5 signalling via JAK-STAT | 9/57 | 8.30E-04 | 3.34E-02 |
| Immune response: MIF - the neuroendocrine-macrophage connector | 8/46 | 8.33E-04 | 3.34E-02 |
| Immune response: Oncostatin M signalling via JAK-STAT in mouse cells | 5/18 | 8.92E-04 | 3.34E-02 |
