# Supplementary material for: Overcoming acquired resistance to HSP90 inhibition by targeting JAK-STAT signalling in triple-negative breast cancer
Source: BMC Cancer. 2019 Jan 24;19:102. doi: 10.1186/s12885-019-5295-z (PMC6345040; doi:10.1186/s12885-019-5295-z)
Supplement: Supplementary file 2 — Figure S1. Positive correlation of pSTAT3 expression with HSP90i sensitivity. A scatter plot displaying the relationship between ganetespib sensitivity (IC50) and pSTAT3 expression in the panel of TNBC cell lines. (DOCX 25 kb) [file 12885_2019_5295_MOESM2_ESM.docx]

**Fig. S1: Positive correlation of pSTAT3 expression with HSP90i sensitivity**

The scatter plot displays the relationship between ganetespib sensitivity (IC_50_) and pSTAT3 expression in the panel of TNBC cell lines. The pSTAT3 protein levels for each cell line were calculated relative to the expression in Hs578T cells. The error bars indicate SEM from three independent experiments. Linear regression analysis was performed, where r^2^ = 0.3766 and p-value < 0.001. The dotted lines indicate the 95% confidence intervals.
