# Supplementary material for: Overcoming acquired resistance to HSP90 inhibition by targeting JAK-STAT signalling in triple-negative breast cancer
Source: BMC Cancer. 2019 Jan 24;19:102. doi: 10.1186/s12885-019-5295-z (PMC6345040; doi:10.1186/s12885-019-5295-z)
Supplement: Supplementary file 3 — Table S2. Compounds that were selectively cytotoxic to parental Hs578T cells compared with HSP90i-resistant clone CR3. Cell viability was assessed after 72 h exposure to a 326-compound small molecule library (1 μM each compound. SELLECK). Z-scores ≤ − 2 identified compounds that were selectively cytotoxic to parental Hs578T cells. (DOCX 13 kb) [file 12885_2019_5295_MOESM3_ESM.docx]

**Table S2: Compounds that were selectively cytotoxic to parental Hs578T cells compared with HSP90i-resistant clone CR3**

|  | **Compound** | **Target** | **Z-score** |
| --- | --- | --- | --- |
| 1 | AT9283 | BCR-ABL/JAK/Aurora Kinase | -7.35 |
| 2 | Mitoxantrone | Topoisomerase | -5.63 |
| 3 | Obatoclax Mesylate | BCL2 | -4.31 |
| 4 | Camptothecin | Topoisomerase | -4.23 |
| 5 | SNS-032 (BMS-387032) | CDK | -4.06 |
| 6 | AT7519 | CDK | -3.82 |
| 7 | PHA-739358 (Danusertib) | Aurora Kinase/BCR-ABL | -3.61 |
| 8 | PHA-793887 | CDK | -3.47 |
| 9 | ZM-447439 | Aurora Kinase | -3.13 |
| 10 | 2-Methoxyestradiol | HIF | -2.88 |
| 11 | Adriamycin | Topoisomerase | -2.72 |
| 12 | GSK461364 | PLK | -2.71 |
| 13 | GSK1059615 | PI3K/MTOR | -2.68 |
| 14 | AZD1152-HQPA (Barasertib) | Aurora Kinase | -2.67 |
| 15 | PHA-680632 | Aurora Kinase | -2.60 |
| 16 | Deforolimus (MK-8669) | MTOR | -2.43 |
| 17 | Topotecan Hydrochloride | Topoisomerase | -2.13 |
| 18 | AZD7762 | CHEK | -2.10 |
| 19 | Dasatinib | SRC/ABL | -2.09 |
| 20 | VX-680 | Aurora Kinase | -2.08 |
| 21 | Ganetespib | HSP90 | -1.90 |
