# Supplementary material for: Overcoming acquired resistance to HSP90 inhibition by targeting JAK-STAT signalling in triple-negative breast cancer
Source: BMC Cancer. 2019 Jan 24;19:102. doi: 10.1186/s12885-019-5295-z (PMC6345040; doi:10.1186/s12885-019-5295-z)
Supplement: Supplementary file 4 — Table S3. Compounds that were differentially cytotoxic to HSP90i-resistant clone CR3 in the presence of ganetespib. Cell viability was assessed after 72 h exposure to a 326-compound small molecule library (1 μM each compound. SELLECK) in the presences or absence of ganetespib (10 nM). Z-scores ≤ − 2 identified compounds that were selectively cytotoxic CR3 cells in the absence of ganetespib, Z-scores ≥2 identified compounds that were selectively cytotoxic CR3 cells in the presence of ganetespib. (DOCX 23 kb) [file 12885_2019_5295_MOESM4_ESM.docx]

**Table S3: Compounds that were differentially cytotoxic to HSP90i-resistant clone CR3 in the presence of ganetespib**

|  | **Compound** | **Target** | **Z-score** |
| --- | --- | --- | --- |
| 1 | Aurora A Inhibitor I | Aurora Kinase | 4.91 |
| 2 | MLN8237 | Aurora | 3.73 |
| 3 | LY2784544 | JAK2 | 3.67 |
| 4 | LY2228820 | p38 MAPK | 2.71 |
| 5 | ENMD-2076 | FLT/Aurora Kinase/SRC/VEGFR | 2.64 |
| 6 | Tipifarnib | Farnesyltransferase (Ftase) | 2.63 |
| 7 | Phleomycin | DNA | 2.44 |
| 8 | U0126-EtOH | MEK | 2.35 |
| 9 | SB 431542 | ALK | 2.11 |
| 10 | PHA-739358 | Aurora Kinase/BCR-ABL | 2.09 |
| … | ………………… | …………………… | ……….. |
| 324 | BEZ235 | PI3K and mTOR | -2.15 |
| 325 | NPI-2358 | Beta tubulin | -2.30 |
| 326 | ITF2357 | Class I and II HDACs | -2.35 |
| 327 | Ispinesib | KSP | -3.24 |
| 328 | SB 743921 | KSP (Eg5) | -3.55 |
